# Additive Manufacturing of Gear Electrodes and EDM of a Gear Cavity

**DOI:** 10.3390/mi16101153

**Published:** 2025-10-11

**Authors:** Kai Jiang, Yangquan Liu, Bin Xu, Shunda Zhan, Junwei Liang

**Affiliations:** 1 School of Computer Science and Software Engineering, Shenzhen University of Information Technology, Shenzhen 518172, China; jiangkai@sziit.edu.cn; 2 Shenzhen Key Laboratory of High Performance Nontraditional Manufacturing, Shenzhen University, Shenzhen 518060, China; yangquanliu@szu.edu.cn (Y.L.);; 3 School of Intelligent Manufacturing and Equipment, Shenzhen University of Information Technology, Shenzhen 518172, China; zhanshunda@sziit.edu.cn

**Keywords:** selective laser sintering, electrode gear cavity, conductance, EDM

## Abstract

Plastic gears are used in a variety of industrial fields and are primarily produced by injection molding with a gear cavity. At present, EDM is usually used for machining gear cavities with metal materials, and the tool electrode used in the process is usually machined through a milling process. For helical gear cavities and helical bevel gear cavities, certain problems are encountered when the tool electrodes of EDM are obtained from milling procedures, including waste of raw materials and the complex technical process. Focusing on the above problems, this paper used copper powder to fabricate gear electrodes through a selective laser sintering process. The obtained gear electrodes underwent heat treatment and the effects of the main process parameters on the electrical conductance of tool electrodes were analyzed in this study. Finally, the heat-treated gear electrodes were applied to EDM to fabricate a helical gear cavity and a helical bevel gear cavity. During EDM, the TWR and MRR of the gear electrodes were 0.0029 mm^3^/min and 0.3872 mm^3^/min, respectively. Compared with that of gear electrodes made by the milling process, the MRR of the gear electrodes fabricated by SLS improved by 31.53%.

## 1. Introduction

Plastic gears are widely used in auto parts, micro-motors, electronic products and so on [1]. They are usually mass-produced by injection molding with gear cavities [2,3]. At present, electric discharge machining (EDM) is usually used for machining gear cavities with metal materials [4,5]. EDM is a machining process which can remove workpiece material through electrical discharge [6,7]. Therefore, EDM is suitable for machining parts from metal materials with high hardness, high strength and high brittleness [8,9,10].

The tool electrode used in the EDM of gear cavities is usually produced by the milling process. For helical gear cavities and helical bevel gear cavities, due to their complex structural characteristics, tool electrodes fabricated by the milling process have certain disadvantages. These disadvantages include waste of raw materials, a long machining cycle and a complex technical process [11,12]. Copper was selected for this study as it is a representative and widely used EDM electrode material, ensuring the practical relevance of the findings. Using copper powder as a raw material, tool electrodes with complex structural characteristics can be manufactured through the selective laser sintering process (SLS), which has advantages of saving raw materials, good production flexibility and high processing efficiency [13,14,15].

Through SLS, parts can be obtained by laser sintering powder, and the properties of parts depends on the type of metal powder [16,17,18]. In EDM, the electrical conductance of the tool electrode has an important influence on the material removal rate (MRR) and tool wear ratio (TWR) [19]. Through SLS, Amorim et al. [16] used copper-based alloy powder to fabricate EDM electrodes and researched their MRR. Using ZrB2-CuNi powders, Czelusniak et al. [20] fabricated an electrode and evaluated its EDM performance. Through SLS, Sahu et al. [21] used metal matrix composite powders to fabricate an electrode and applied it in the EDM of titanium. In order to obtain an electrode with complex geometry, Monzón et al. [22] combined rapid prototyping with electroforming technology to fabricate complex EDM electrodes. Equbal et al. [23] used fused deposition modeling (FDM) process to fabricate a tool electrode and they investigated the EDM performance of the FDM electrode based on the measurement data of MRR, TWR and Ra. Based on the atomic diffusion additive manufacturing (ADAM) process, Bordón et al. [24] used copper powders to fabricate an EDM electrode and they found that the ADAM electrode had a similar material removal rate and Ra to the conventional electrodes.

In EDM, the resistivity of the tool electrode has an important effect on its EDM performance. Grain boundaries and dislocations of grains have important effects on the resistivity of metals [17]. Therefore, tool electrodes obtained by SLS need to be heat-treated to regulate their resistivity and electrical conductance. Through electron back-scatter diffraction methods (EBSD), Hung et al. [25] studied the mechanical properties of annealed wire in detail. Based on the experimental results from EBSD, Konkova et al. [26] researched the influence of annealing treatment on the physical properties of rolled copper. Li et al. [27] studied the microscopic properties of the deformation region in copper material under different heat treatment experiments with the temperature ranging from 200 °C to 800 °C. Based on the EBSD results of cold-drawn copper wire, Brisset et al. [28] researched the effects of the heat-treated parameters on the evolution of microscopic metallography.

In this paper, gear electrodes were fabricated by using SLS, and the electrical conductance of the gear electrodes was then regulated by the heat treatment process, so as to effectively ensure the EDM performance of the copper electrodes.

## 2. Materials and Methods

In this paper, spherical copper powder with an average diameter of 40 μm was sintered by SLS and thus the gear electrodes were obtained. The elemental composition of the copper powder is shown in Table 1. In order to ensure the gear electrodes had the same mechanical properties, the gear electrodes were manufactured under the same SLS parameters [29]. In the SLS of the gear electrodes, the laser power was set to 200 W, the scanning speed of the laser was set to 200 mm/s and each layer thickness of copper powder was set to 30 μm. The substrate plate was preheated to 100 °C [30]. This established parameter set was utilized to ensure consistent fabrication of the complex electrode geometry, thereby allowing the effects of the subsequent heat treatment to be isolated and studied as the primary variable. The gear copper electrodes obtained by SLS were heat-treated in a vacuum furnace at temperatures of 100 °C, 300 °C and 600 °C, respectively, with holding times of 1 h, 2 h and 4 h, respectively. These parameter levels were selected to cover critical microstructural transformation stages (recovery, recrystallization, and grain growth), and they proved sufficient to identify a clear optimum for the EDM performance. After the heat treatment was completed, the electrodes were taken out and their electrical conductance was measured using a conductance meter (Sigma 2008C, Xiamen Tianyan Instruments Co., Ltd., Xiamen, China).

The gear copper electrode was installed on an ultra-precision EDM machine (Sodick, Kaga, Japan). S136 mold steel was used as the workpiece material to carry out the EDM for fabricating the gear cavities. In the EDM of the gear cavity, the peak current, open circuit voltage, pulse width (Ton) and pulse interval (Toff) were set to 4 A, 120 V, 150 μs and 50 μs. The surface topography of the gear cavity was observed using a scanning electron microscope (FEG450, Pleasanton, CA, USA), while its overall topography and surface roughness were observed on a laser confocal microscope (VK-X250, Osaka, Japan).

**Figure 1:**
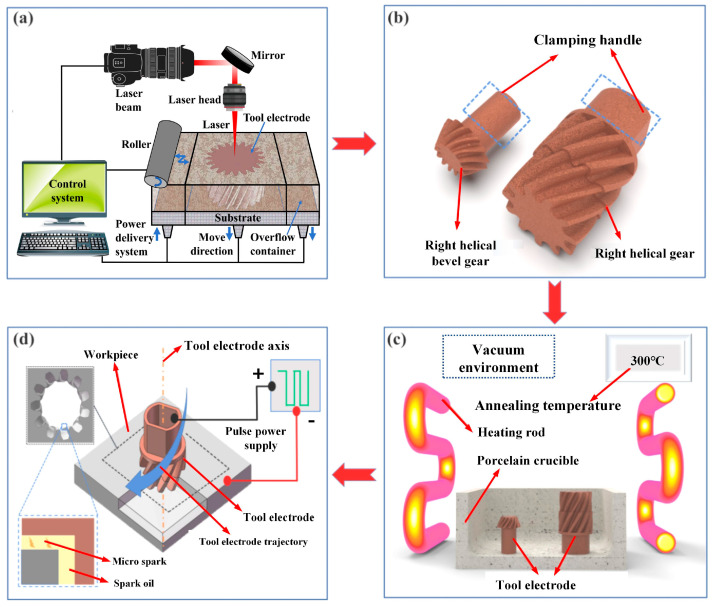
The technological process adopted in this paper. (**a**) Schematic of SLS; (**b**) Tool Electrodes Fabricated by SLS; (**c**) Heat Treatment of Tool Electrodes; (**d**) Schematic of EDM for Gear Cavities.

Figure 1 shows the process flow adopted in this paper. (1) Gear electrodes were designed based on the gear cavity. After that, the gear electrodes were fabricated by SLS using copper powder as raw material (Figure 1a). (2) The electrodes (Figure 1b) were placed in a vacuum furnace for heat treatment (Figure 1c), thereby regulating their conductance and hardness. (3) The electrodes obtained by the above process were clamped on the EDM machine and the process parameters were set. Through the linkage between the *Z*-axis movement and the rotating shaft of the EDM machine, the machining of the gear cavity was carried out (Figure 1d).

## 3. Results and Discussion

The Gaussian distribution of laser energy will lead to uneven heating of copper powder and uneven cooling of the molten pool. Under the influence of the above factors, there can be a lot of dislocation inside the gear electrode [31]. These dislocations will hinder the movement of electrons, resulting in low material electrical conductance [32]. In EDM, the electrical conductance of the tool electrodes is closely related to their EDM performance. The greater the electrical conductance, the smaller the TWR. Focusing on the above problems, the dislocations inside the gear electrode were eliminated by means of heat treatment, so as to regulate electrical conductance.

### 3.1. Effect of the Annealing Temperature on Electrical Conductance

**Figure 2:**
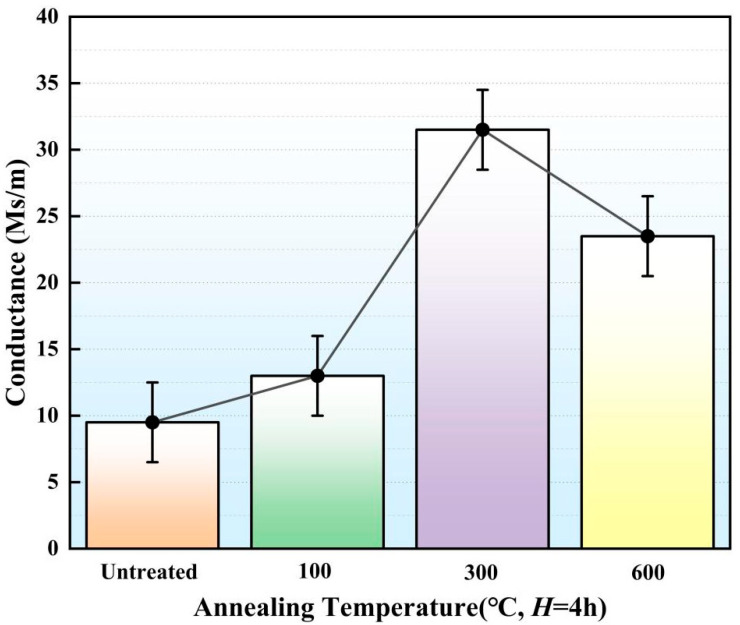
Electrical conductivity of gear electrode after holding for 4 h at different annealing temperatures.

The gear electrodes fabricated by SLS were placed in a vacuum furnace for heat treatment at annealing temperatures (*T*) of 100 °C, 300 °C and 600 °C, respectively. The holding time (*H*) was set to 4 h. After heat treatment, the electrical conductance of the gear electrode was tested (Figure 2). The electrical conductance of the gear electrode that did not undergo heat treatment was 9.51 Ms/m. When the *T* was equal to 100 °C, the conductance was 12.91 Ms/m. As the *T* was increased to 300 °C, the electrical conductance increased to 33.8 Ms/m. When the *T* was equal to 600 °C, the electrical conductance decreased to 23.07 Ms/m. The above experimental results showed that the heat treatment process can eliminate internal dislocation of the gear electrode, thus promoting the electrical conductance.

**Figure 3:**
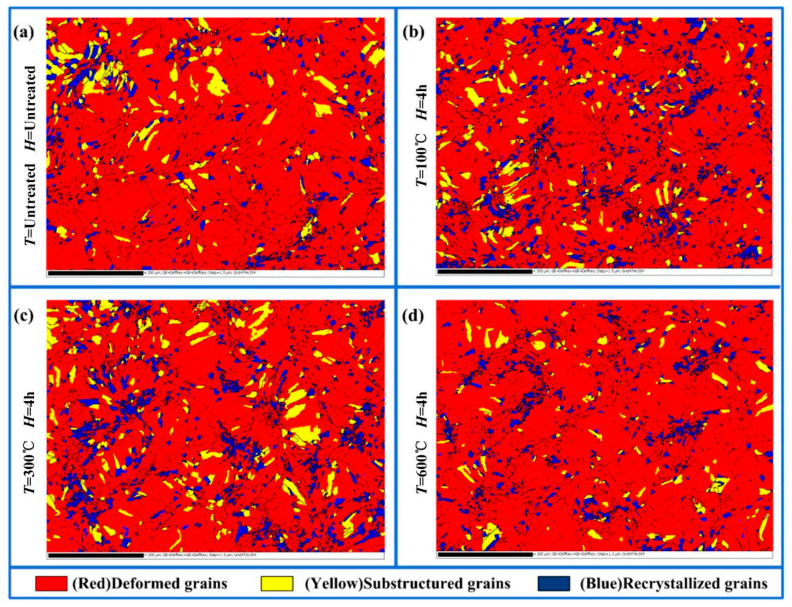
DefRex diagram of gear electrode under different heat treatment parameters. (**a**) Untreated; (**b**) Annealing temperature *T* = 100 °C, holding time *H* = 4 h; (**c**) Annealing temperature *T* = 300 °C, holding time *H* = 4 h; (**d**) Annealing temperature *T* = 600 °C, holding time *H* = 4 h.

The gear electrode that did not undergo heat treatment had lots of dislocations and high dislocation density. In this case, the dislocations were intertwined to form dislocation walls. Under the influence of *T*, dislocation walls would further develop into sub-grains. With further heat treatment, the sub-grain boundaries continued to absorb dislocations and dynamically recrystallized, finally forming high-angle grain boundaries (HABs). A DefRex diagram is obtained by electron back-scattered diffraction (EBSD) calculation, which can observe the recrystallization state [33]. In the DefRex diagram, the different colored regions represented the different morphologies of grains. The recrystallized grains are marked in the blue regions, the substructural grains are marked in the yellow regions and the deformed grains are marked in the red regions. The DefRex diagrams of gear electrodes under different heat treatment parameters are shown in Figure 3. As shown in Table 2, the recrystallization ratio of the gear electrode that did not undergo heat treatment was 7.26% (Figure 3a). As the *T* increased from 100 °C to 300 °C, the recrystallization ratio of the gear electrode increased from 11.65% (Figure 3b) to 12.41% (Figure 3c). The above experimental results showed that the increase in *T* could promote recrystallization and lead to a decrease in dislocation density. Therefore, with the *T* increasing from 100 °C to 300 °C, the electrical conductance increased from 9.51 Ms/m to 33.8 Ms/m.

With the *T* increasing from 300 °C to 600 °C, the recrystallization ratio of the gear electrode decreased from 12.41% to 7.39% (Figure 3d) while the small grains fused together to form larger grains. Therefore, based on the EBSD results, the average grain size showed increasing trends with quantitative values from 11.82 μm to 12.95 μm. When the *T* increased from 300 °C to 600 °C, the proportion of deformed grains increased from 78.66% to 88.21% (Table 2). The above experimental results showed that a continued increase in the *T* could effectively form large grains and deformed grains. The deformed grains were full of dislocations, which eventually led to the decreased electrical conductance of the electrode (Figure 2).

### 3.2. Effect of Holding Time on Electrical Conductance

**Figure 4:**
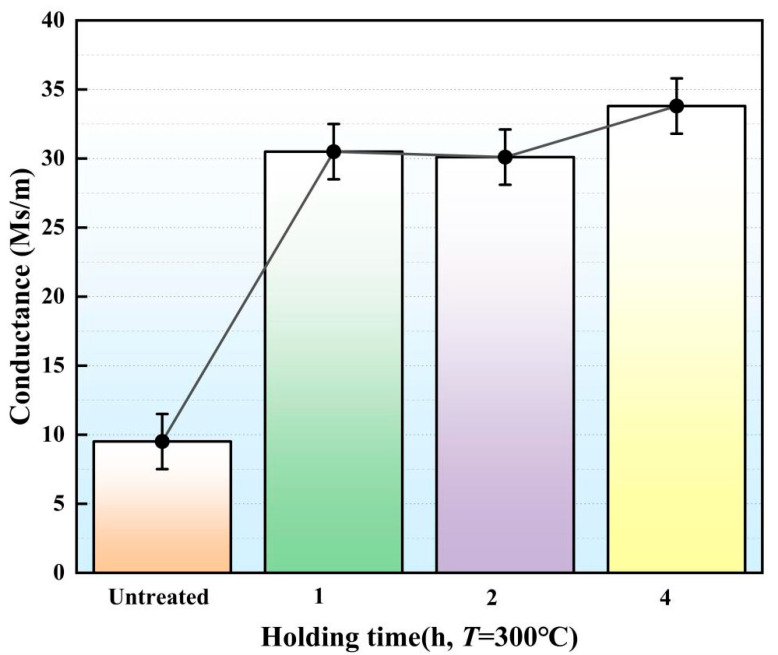
The electrical conductivity of the gear electrode after holding at *T* = 300 °C for different amounts of time.

Under the action of *T*, holding time (*H*) can ensure the formation of sub-grains and large grain boundaries and lead to changes in electrical conductance of the gear electrode. The electrodes fabricated for this experiment underwent heat treatment at a temperature *T* of 300 °C, with holding times set to 1 h, 2 h and 4 h. After heat treatment, the electrical conductance of the gear electrode was tested (Figure 4). When the *H* was 1 h, the electrical conductance was 30.5 Ms/m. When the *H* was 2 h, the electrical conductance decreased to 30.1 Ms/m. When the *H* was 4 h, the electrical conductance increased to 33.8 Ms/m.

**Figure 5:**
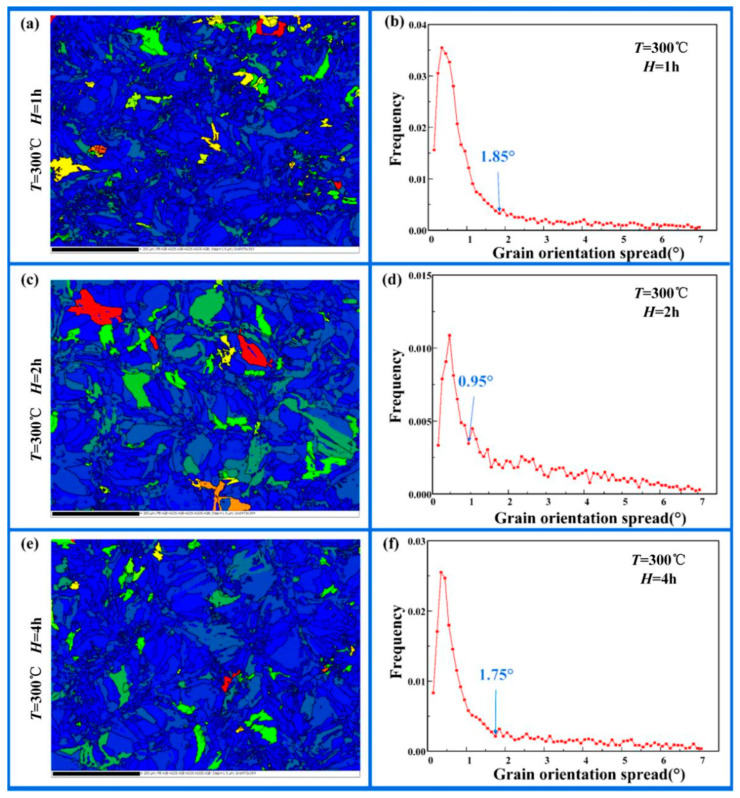
The grain orientation distribution (GOS) of the gear electrodes under different heat treatment parameters: (**a**) *T* = 300 °C, *H* = 1 h; (**b**) the GOS value line diagram of Figure 5a; (**c**) *T* = 300 °C, *H* = 2 h; (**d**) the GOS value line diagram of Figure 5c; (**e**) *T* = 300 °C, *H* = 4 h; (**f**) the GOS value line diagram of Figure 5e.

Grain orientation spread (GOS) is an important method for determining grain distortion [34,35,36]. In the GOS diagram, red and yellow indicate serious grain distortion in a particular region, with a higher dislocation density found there. The critical GOS value is the last point of the first peak. As shown in Figure 5, these values were found to be 1.85°, 0.95° and 1.75° at holding times of 1 h, 2 h and 4 h. When the GOS value of the grain is less than the critical GOS value, the dislocation density inside the grain can be considered to be low [37]. According to the experimental results, when the holding times were 1 h, 2 h and 4 h, the proportion of grains with the GOS value less than the critical value was 79.6%, 44.1% and 70.9%, respectively. Therefore, the electrical conductance of the gear electrode first decreased and then increased with an increased *H* from 1 h to 4 h (Table 3).

In addition, with the *H* increasing from 1 h to 2 h, the proportion of deformed grains increased from 80.93% to 91.64% (Table 2) and the average grain size in gear electrode increased from 10.57 μm to 14.12 μm. In the above process, small grains fused and grew into larger grains, while the recrystallization ratio decreased. With an increase in deformed grains, the dislocation density also increased, which eventually led to a decrease in electrical conductance (Figure 4). With the *H* increasing from 2 h to 4 h, the average grain size in the gear electrode decreased from 14.12 μm to 11.82 μm, while the proportion of deformed grains decreased from 91.64% to 78.66% (Table 2). In the above process, the dislocations in the deformed grains formed sub-grains and further underwent dynamic recrystallization. The formation of sub-grains and dynamic recrystallization absorbed dislocations, resulting in a decrease in the dislocation density. Therefore, under the influence of the above factors, the electrical conductance of the gear electrode tended to increase.

### 3.3. EDM Performance of the Gear Electrode

**Figure 6:**
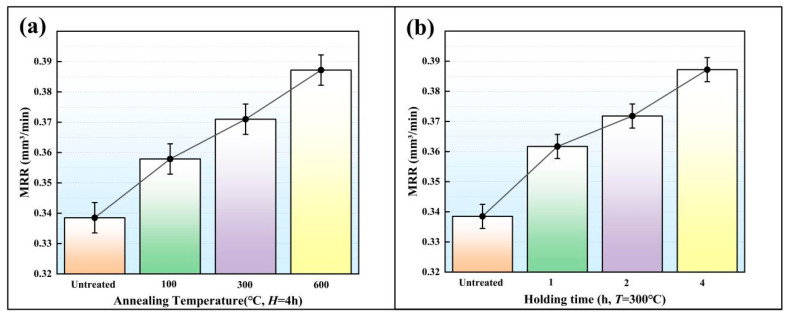
The average MRR values under different heat treatment parameters. (**a**) MRR at Different Annealing Temperatures while *H* = 4 h; (**b**) MRR at Different Holding Times while *T* = 300 °C.

**Figure 7:**
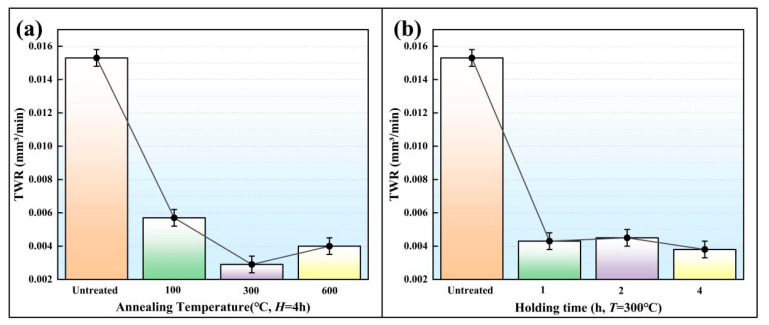
The average TWR values under different heat treatment parameters. (**a**) TWR at Different Annealing Temperatures while *H* = 4 h; (**b**) TWR at Different Holding Times while *T* = 300 °C.

The gear electrodes were placed in a vacuum furnace to carry out the heat treatment under different *T* values of 100 °C, 300 °C and 600 °C. The *H* was set to 1 h, 2 h and 4 h. Nine heat-treated gear electrodes were used in the EDM. The voltage, peak current, Ton and Toff were respectively set to 120 V, 4 A, 150 μs and 50 μs. After EDM, the MRR and the TWR were measured (Figure 6 and Figure 7). According to Figure 6 and Figure 7, the MRR and TWR of the SLS electrode are mainly affected by *T*, while the *H* has a slight influence.

MRR is an important index for measuring the efficiency of EDM. As shown in Figure 6a, the MRR values under a specific *T* and different *H* were averaged to obtain the influence of the *T* on MRR. In Figure 6b, the MRR values under a specific *H* and different *T* were weighted averaged to obtain the influence of the *H* on MRR. As shown in Figure 6, MRR can be improved most effectively by heat preservation at 600 °C for 4 h. The MRR of the gear cavity obtained by EDM without heat treatment was 0.3385 mm^3^/min. Under the impact of *T* and *H*, the MRR was improved, with the value varying from 0.3579 mm^3^/min to 0.3872 mm^3^/min. Some defects, such as pores and micro-cracks, were observed in the gear electrode that did not undergo heat treatment. These defects had an adverse impact on the MRR and the EDM performance of the electrodes. The heat treatment process can eliminate pores and micro-cracks, thus improving both the EDM performance of the gear electrode and the MRR.

**Figure 8:**
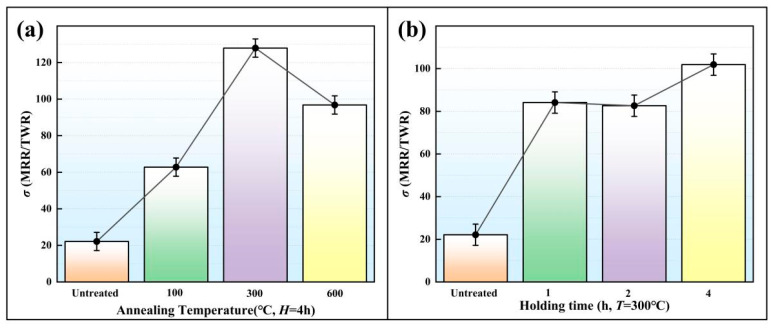
The average *σ* values under different heat treatment parameters. (**a**) *σ* at Different Annealing Temperatures while *H* = 4 h; (**b**) *σ* at Different Holding Times while *T* = 300 °C.

In EDM, TWR is an important index to measure the tool electrode wear. The greater the TWR, the greater the wear. Figure 7 shows the average TWR values under different heat treatment parameters. The TWR values under a specific *T* and different *H* were averaged to obtain the influence of the *T* on TWR (Figure 7a). The TWR values under a specific *H* and different *T* were weighted-averaged to obtain the influence of the *H* on TWR (Figure 7b). *σ* is the ratio of MRR to TWR. The larger the *σ*, the higher the cost performance of the EDM. The average *σ* values under different heat treatment parameters are shown in Figure 8. As shown in Figure 6, the MRR of the gear cavity did not change much. In order to obtain a higher *σ* and ensure the effectiveness of the EDM, it is first necessary to reduce the TWR of the gear electrode, a property which is closely related to the electrical conductance. The greater the electrical conductance of the gear electrode, the smaller the TWR.

As shown in Figure 4, under the effect of *T* = 300 °C and *H* = 4 h, the gear electrode displayed its maximum electrical conductance (31.5 Ms/m). At this time, as shown in Figure 7, the gear electrode showed the smallest TWR (0.0029 mm^3^/min) for this particular annealing parameter. Under this working condition, as shown in Figure 8, EDM also showed the largest *σ* (128). When the *T* and the *H* were respectively set to 100 °C and 4 h, the gear electrode showed its minimum electrical conductance value (12.23 Ms/m, Figure 4), and the gear electrode displayed its maximum TWR (0.006 mm^3^/min, Figure 7). Under this working condition, as shown in Figure 8, the EDM also displayed the smallest value of *σ* (60).

## 4. EDM of Gear Cavity

For verifying the feasibility of the proposed process, helical gear electrodes and helical bevel gear electrodes were designed. The modulus for the helical gear electrode was 1, the number of teeth was 11 and the helical angle was 30°. The modulus for the helical bevel gear was 0.7, the number of teeth was 13 and the helical angle was 30°. Helical gear electrodes and helical bevel gear electrodes were fabricated by SLS. Then, the obtained gear electrodes were heat-treated with *T* = 300 °C and *H* = 4 h. The voltage, *T_on_* and *T_off_* were set to 120 V, 150 μs, and 50 μs respectively. After that, the helical gear electrode and helical bevel gear electrode were used in the EDM of the gear cavity.

**Figure 9:**
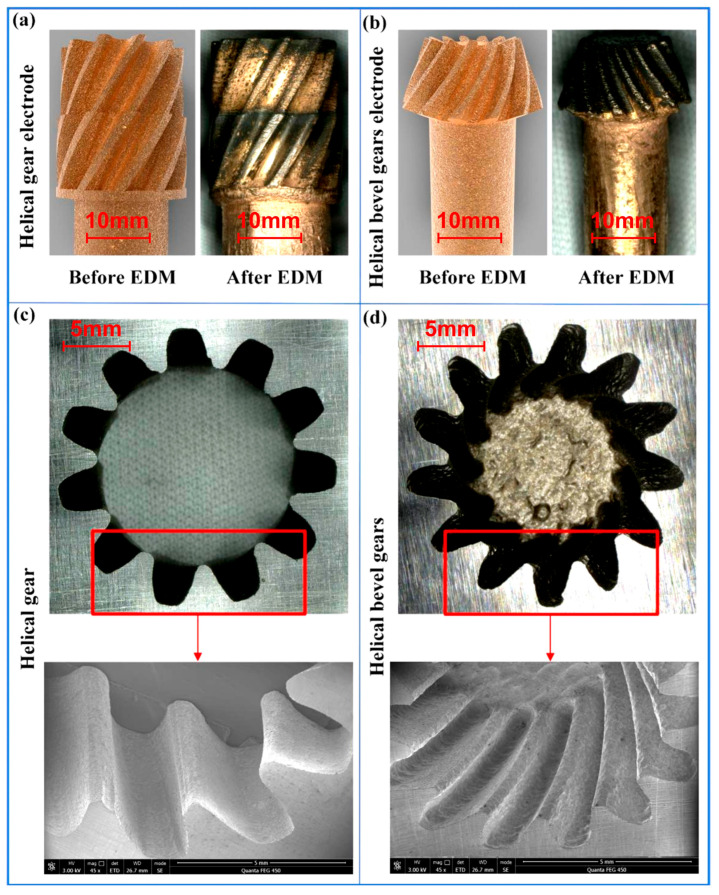
(**a**) The helical gear electrode before EDM (images from CAD) and after EDM; (**b**) the helical bevel gear electrode before EDM (images from CAD) and after EDM; (**c**) the obtained helical gear cavity; (**d**) the obtained helical bevel gear cavity.

The surface topography and shape accuracy of the helical gear cavity and the helical bevel gear cavity were satisfactory (Figure 9). The surface roughness (Ra) of the gear cavity was 2.43 μm. The gear electrode obtained by the milling process was also applied in the EDM of the gear cavity and its MRR value was 0.2845 mm^3^/min. When the gear electrode made by SLS was applied in EDM, the MRR value was 0.3742 mm^3^/min. The above experimental results showed that the EDM efficiency of the gear cavity can be effectively improved by using the gear electrode fabricated by SLS.

## 5. Conclusions

For helical gear cavities and the helical bevel gear cavities, due to their complex structural characteristics, tool electrodes fabricated by the milling process have certain disadvantages. These disadvantages include waste of raw materials, a long machining cycle and a complex technical process. The comparison with conventional milling was strategically centered on the pivotal EDM performance indicators (MRR and TWR) to underscore the practical feasibility and efficiency of the SLS-based approach for complex geometries.

(1) The gear electrode fabricated by SLS displayed a lot of dislocations, resulting in its low electrical conductance. The heat treatment process can eliminate the dislocations of the gear electrode, thus effectively improving the electrical conductance. Compared with *H*, *T* can significantly affect the electrical conductance of the gear electrode. When the *T* = 300 °C and *H* = 4 h, the gear electrode displayed its maximum electrical conductance value (33.8 Ms/m).
(2) The heat treatment process showed little effect on the MRR but had a significant impact on the TWR of the gear electrodes. TWR is closely related to the electrical conductance. The greater the conductance, the smaller the TWR. The gear electrode displayed its maximum electrical conductance with *T* of 300 °C and *H* of 4 h. At this time, the gear electrode also had the smallest TWR value (0.0029 mm^3^/min).
(3) Under the effect of 120 V voltage, 150 μs pulse width and 50 μs pulse interval, the gear electrode was used in EDM, obtaining the helical gear cavity and the helical bevel gear cavity with good surface topography and geometric accuracy. The surface roughness (Ra) of the gear cavity was 2.43 μm. Compared with that of the gear electrode obtained by the milling process, the MRR of the gear electrode fabricated by SLS improved by 31.53%, thus effectively improving the EDM efficiency of the gear cavity.

## Figures and Tables

**Table 1:** Elemental composition of copper powder.

Element.	Cu	Si	*p*	Sn	Ni	Nb
Content (%)	99.37	0.43	0.084	0.04	0.036	0.033

**Table 2:** The average grain size, the proportion of recrystallized grains, substructured grains and deformed grains under different heat treatment parameters.

	Untreated	*T =* 100 °C *H* = 4 h	*T =* 300 °C *H* = 1 h	*T =* 300 °C *H* = 2 h	*T =* 300 °C *H* = 4 h	*T =* 600 °C *H* = 4 h
Average grain size (µm)	13.64	11.7	10.57	14.12	11.82	12.95
Recrystallized grains (%)	7.26	11.65	14.25	4.97	12.41	7.39
Substructured grains (%)	7.70	6.14	4.82	3.39	8.93	4.41
Deformed grains (%)	85.04	82.21	80.93	91.64	78.66	88.21

**Table 3:** The electrical conductivity of the gear electrode under different heat treatment parameters.

No.	Annealing Temperature *T* (°C)	Holding Time *H* (h)	Conductance (Ms/m)
1	100	1	13.94
2	300	1	30.50
3	600	1	24.32
4	100	2	12.23
5	300	2	30.10
6	600	2	23.69
7	100	4	12.91
8	300	4	33.80
9	600	4	23.07
10	Untreated	Untreated	9.51

## Data Availability

All data generated or analyzed during this study are included in this published article.
